# LONELINESS IS INVERSELY ASSOCIATED WITH EXERCISE AND SLEEP APNEA IN OLDER WOMEN AT RISK FOR ALZHEIMER’S DISEASE

**DOI:** 10.1093/geroni/igac059.2800

**Published:** 2022-12-20

**Authors:** Madina Danish, Alyx Shepherd, Kitty Lui, Atul Malhotra, Sheri Hartman, Ellen Lee, Erin Sundermann, Sarah Banks

**Affiliations:** University of California San Diego, San Diego, California, United States; University of California, San Diego, San Diego, California, United States; University of California, San Diego, San Diego, California, United States; University of California, San Diego, San Diego, California, United States; University of California, San Diego, San Diego, California, United States; University of California, San Diego, San Diego, California, United States; University of California, San Diego, San Diego, California, United States; University of California, San Diego, San Diego, California, United States

## Abstract

**Background:**

Modifiable risk factors (MRF) for Alzheimer’s disease (AD) include sedentary behavior, sleep apnea, and loneliness; however, how these MRFs influence one another is unclear. We examined correlations among loneliness, physical activity and sleep apnea among older women at higher risk for AD.

**Methods:**

Data were collected as part of the Women: Inflammation and Tau Study, which recruits women age 65–85 with higher AD genetic risk and mild impairment on the Montreal Cognitive Assessment. Participants completed the UCLA Loneliness Scale and home sleep tests to derive the Apnea Hypopnea Index (AHI), a measure of sleep apnea severity. Women wore ActiGraph accelerometers for one week to measure average Moderate-Vigorous Activity per day (MVPA). We used Spearman correlation to examine the relationships among AHI, MVPA and Loneliness Scale score.

**Results:**

Preliminary data were available for 12 women (mean age=72.4 [SD=2.8], 100% non-Hispanic White). MVPA ranged from 0.3–52.8 minutes (mean=9.38 [SD=14.1]; AHI ranged from 1.5–28.3 (mean=14.0 [SD=9.0] and Loneliness Scale score ranged from 0–34 (mean=9.8 [SD=x]). Significant, positive associations were observed between AHI and Loneliness (rho=.80, p=.006) and a moderate relationship at trend level was notable between higher MVPA and less Loneliness (rho=-9.53, p=.07). AHI did not relate to MVPA.

**Conclusion:**

Results suggest that loneliness independently relate to both sleep apnea severity and sedentary behavior in older women. Combined interventions that target loneliness in addition to physical activity and sleep may be important in women at-risk for AD.

